# Pathophysiological role of calcium channels and transporters in the multiple myeloma

**DOI:** 10.1186/s12964-021-00781-4

**Published:** 2021-09-27

**Authors:** Tingting Li, Junmin Chen, Zhiyong Zeng

**Affiliations:** 1 Department of Hematology, The First Affiliated Hospital of Fujian Medical University, 20 Chazhong Road, Fuzhou, Fujian 350005 People’s Republic of China; 2 Fujian Key Laboratory of Laboratory Medicine, Fuzhou, People’s Republic of China

**Keywords:** Plasma membrane Ca^2+^ transporters, Store-operated Ca^2+^ entry, Mitochondrial Ca^2+^ transporters, Myeloma bone disease, Prognosis

## Abstract

Multiple myeloma (MM) is a common malignant tumor of plasma cells. Despite several treatment approaches in the past two decades, MM remains an aggressive and incurable disease in dire need of new treatment strategies. Approximately 70–80% of patients with MM have myeloma bone disease (MBD), often accompanied by pathological fractures and hypercalcemia, which seriously affect the prognosis of the patients. Calcium channels and transporters can mediate Ca^2+^ balance inside and outside of the membrane, indicating that they may be closely related to the prognosis of MM. Therefore, this review focuses on the roles of some critical calcium channels and transporters in MM prognosis, which located in the plasma membrane, endoplasmic reticulum and mitochondria. The goal of this review is to facilitate the identification of new targets for the treatment and prognosis of MM.

**Video Abstract**

**Supplementary Information:**

The online version contains supplementary material available at 10.1186/s12964-021-00781-4.

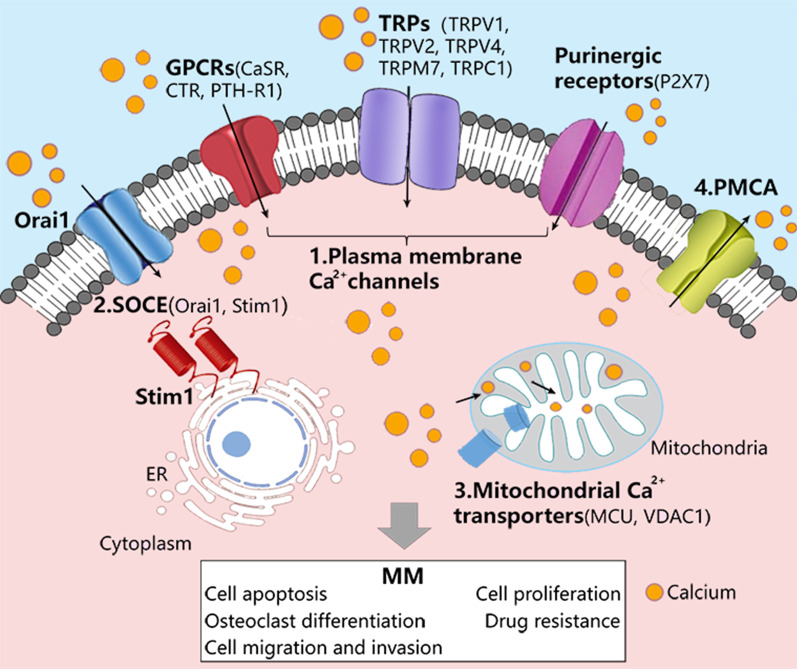

## Introduction

Multiple myeloma (MM), a malignant tumor with abnormal accumulation of terminally differentiated plasma cells, is the second most common hematologic cancer after lymphoma [1, 2]. Most patients with MM will have varying degrees of bone destruction [3], renal insufficiency and hypercalcemia [4], which is closely related to calcium regulation. Studies have confirmed that serum calcium level reflects the ability of bone resorption and bone formation [5]. Hypercalcemia is an adverse prognostic factor in MM [6], and it is most common in those myeloma patients who have the greatest tumor volume or patients with plasma cell leukemia (a late stage complication of myeloma) [7]. The reasons for this are still unclear, but they may be related to bone resorption activity produced by myeloma cells and glomerular filtration status. Because myeloma patients often have irreversible kidney damage and increased renal tubular calcium reabsorption, resulting in elevated serum calcium concentration and abnormal bone remodeling [7, 8]. In addition, previous studies have reported that the interaction between MM cells and osteoclasts accelerates bone destruction and bone remodeling in myeloma, which leads to an increase in calcium concentration in serum and bone marrow [7, 9]. Therefore, MM cells may be exposed to a high concentration of extracellular calcium in the bone marrow microenvironment, but so far, the relevant calcium transporters/channels and mechanisms of calcium regulation in MM are still unclear.

**Fig. 1:**
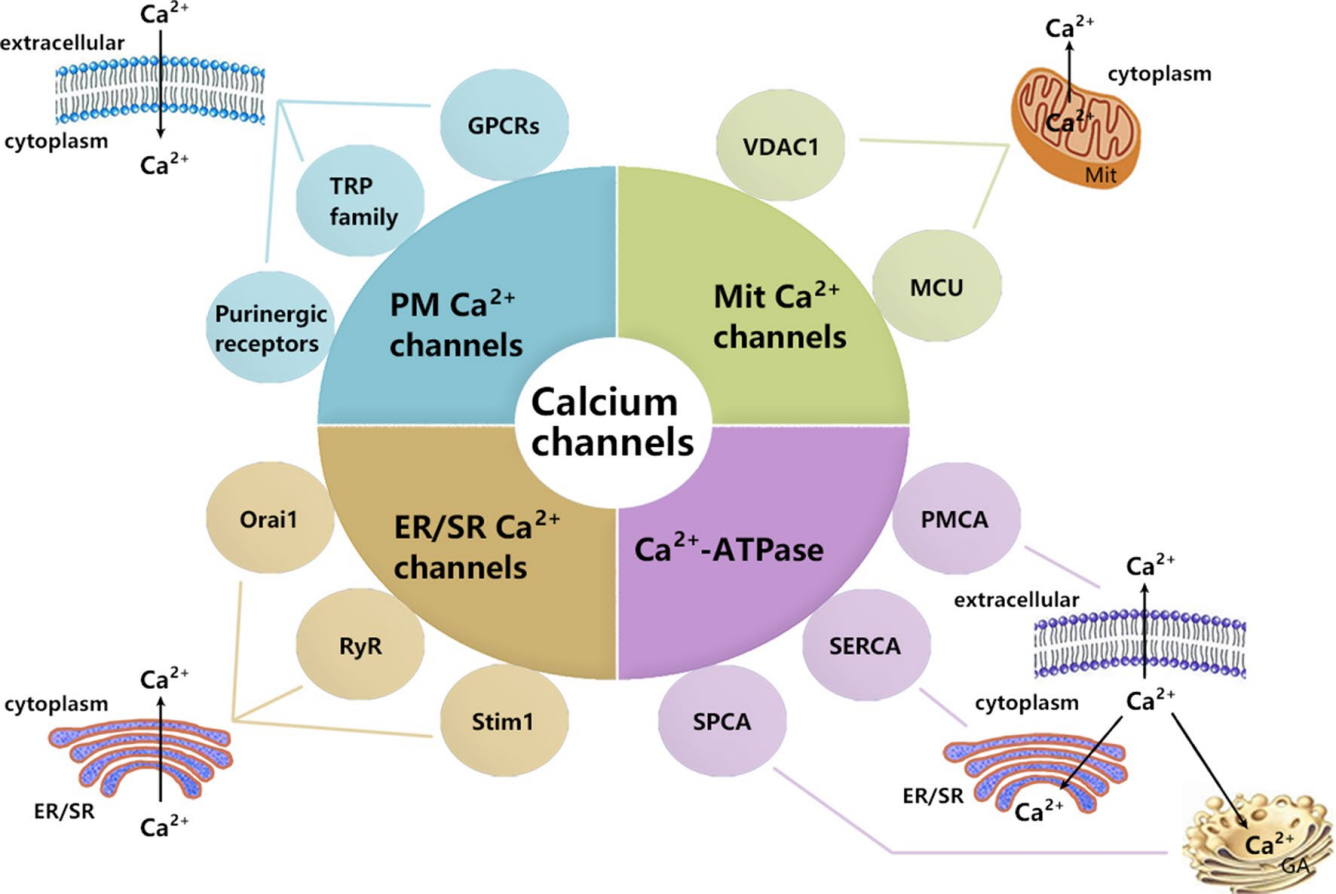
The composition of calcium channels and transporters. PM: plasmic membrane; Mit: mitochondrial; ER/SR: endoplasmic/sarcoplasmic reticulum; GA: Golgi apparatus; TRP: transient receptor potential channel; GPCRs: G protein-coupled receptors; Stim1: Stromal-interaction molecule1; RyR: ryanodine receptor; VDAC1: Voltage-Dependent Anion Channel 1; MCU: Mitochondrial Ca^2+^ uniporter; PMCA: Plasma membrane Ca^2+^-ATPase; SERCA: endoplasmic/sarcoplasmic reticulum Ca^2+^-ATPase; SPCA: secretory pathway Ca^2+^-ATPase

Calcium channels and transporters are protein structures that exist in cell membranes, endoplasmic reticulum membranes, and mitochondrial membranes. They can mediate Ca^2+^ balance inside and outside of the membrane, thus maintaining the physiological functions of the organism. The network control of calcium channels and transporters which regulate intracellular Ca^2+^ homeostasis includes: (1) calcium channels or transporters that allow Ca^2+^ influx from extracellular Ca^2+^ storage across the plasma membrane (PM), such as transient receptor potential channel (TRP), G Protein-Coupled Receptors (GPCRs) and purinergic receptor (ATP-gated cation channel P2X7 receptor); (2) Inositol 1,4,5-triphate receptor (IP3R) or ryanodine receptor (RyR) and stromal-interaction molecule1 (Stim1) combined with the plasma membrane calcium channel protein Orai1 (also known as CRACM1) to mediate Ca^2+^ release from the endoplasmic/sarcoplasmic reticulum (ER/SR); (3) Mitochondrial Voltage-Dependent Anion Channel 1 (VDAC1) and Ca^2+^ uniporter (MCU) regulate mitochondrial Ca^2+^ uptakes; (4) Ca^2+^-ATPase pumps Ca^2+^ from cytoplasm back to ER/SR or extracellular space [10, 11] (Fig. 1). Most features of cancer, if not all, involve calcium signaling to mediate critical cellular processes, including transcriptional regulation, which underlies the gene expression in a variety of pathways essential for tumorigenesis and metastasis [12]. So far, various studies have shown that changes in calcium channels and transporters are related to the proliferation, apoptosis, osteoclast differentiation and outcome of MM. For instance, the knockdown of the non-selective cation channel TRP proteins, known as a type of calcium channel on the cell membrane, was shown to result in the differentiation of osteoclast in MM [13, 14]. Purinergic receptor P2X7 activation induces cell death in human RPMI 8226 multiple myeloma cells [15]. Up-regulation of Stim1 or Orai1 [two critical regulators of Store-Operated Ca^2+^ Entry (SOCE)] was associated with the clinical outcome of MM, and Stim1 or Orai1 down-regulation reduce cell viability, cause cell apoptosis and cell cycle arrest in MM cell lines [16]. In human CD45^+^ U266 myeloma cells, VDAC1 might sensitize to many extracellular stimuli that trigger apoptosis via the mitochondrial pathway [17].

**Fig. 2:**
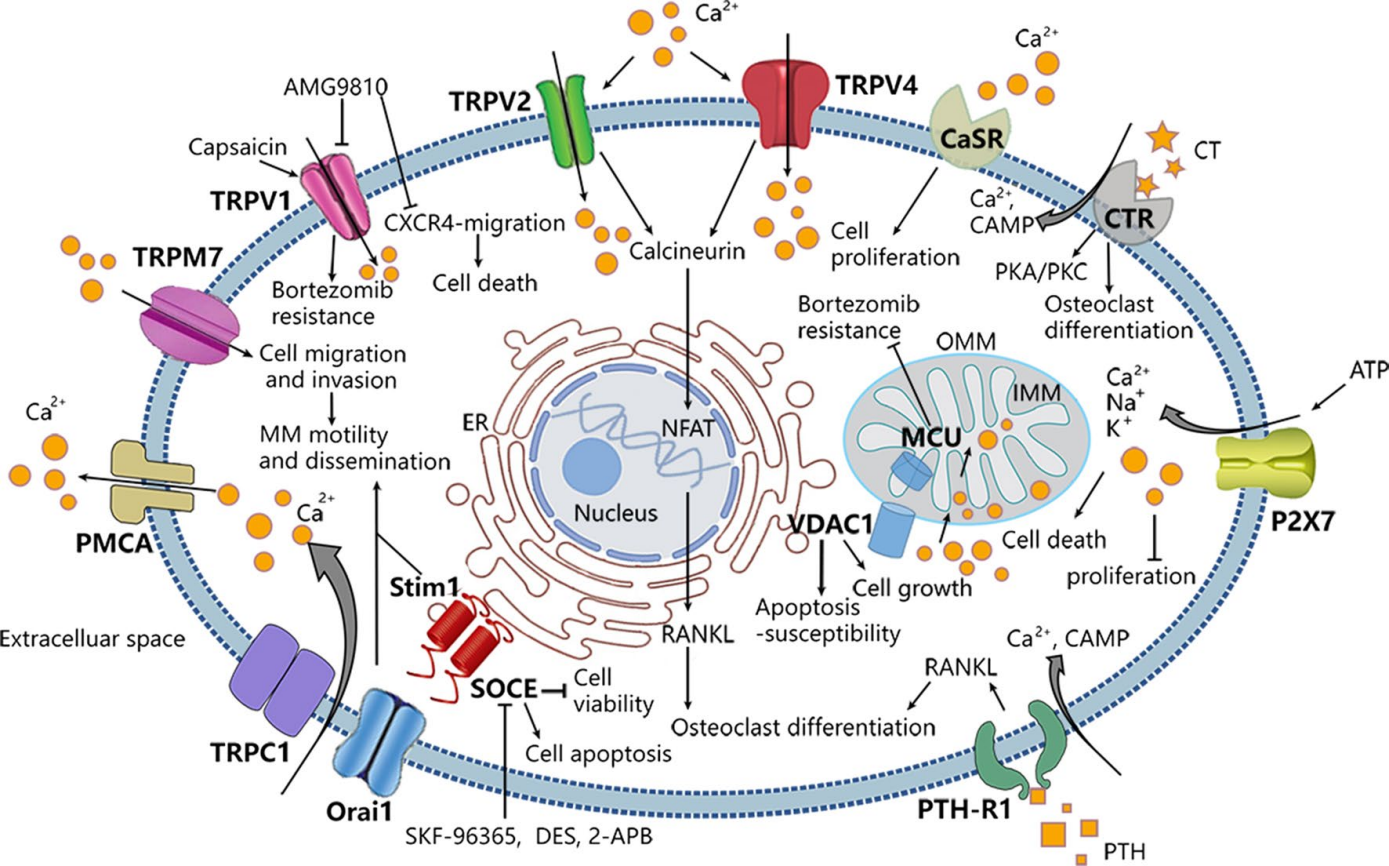
Important Ca^2+^ channels/transporters in multiple myeloma cells. ER: Endoplasmic reticulum; OMM: Outernal mitochondrial membranes; IMM: internal mitochondrial membranes. The intracellular Ca^2+^ is governed by a series of proteins: (1) plasma membrane Ca^2+^ channels or transporters, such as TRPs (TRPV1, TRPV2, TRPV4, TRPM7), G Protein-Coupled Receptors (CaSR, CTR, PTH-R1), Purinergic receptors (P2X7), which mediate Ca^2+^ influx into cells. (2) Store-Operated Ca^2+^ Entry, as one of the major pathways for Ca^2+^ influx across plasma membrane. (3) Mitochondrial Ca^2+^ transporters, including VDAC1 and MCU, mediate Ca^2+^ transport across internal and outernal mitochondrial membranes. (4) Ca^2+^-ATPases pumping Ca^2+^ from cytosol to extracellular space. Ca^2+^ can regulate various cellular events, including gene transcription, proliferation, migration and apoptosis. During development of multiple myeloma, the alteration of Ca^2+^ channels/transporters lead to changes in Ca^2+^ permeability and distribution inside and outside the cell membrane as well as activation of various signaling pathways, providing a suitable microenvironment for the growth of tumor cells. Targeting the dysregulated Ca^2+^ channels/transporters may improve the prognosis of patients with MM

Therefore, in this review, we summarize the expression, localization and pathophysiological role of some essential calcium channels and transporters, including the transient receptor potential channel (TRP) family, GPCR family, Purinergic receptors, SOCE channels and Mitochondrial Ca^2+^ transporters, which have been reported to be altered in MM (Fig. 2, Table 1).

**Table 1:** Expression, localization and phthophysiological function of calcium channels and transporters in MM

Name	Related channels/transporters	Main localization	Compound	Mechanism	Pathophysiological role in MM	References
TRPVs	TRPV1	Plasma membrane	Capsaicin	Activator	TRPV1 inhibitor has synergistic anti-MM activity with bortezomib	[47]
	TRPV2	Plasma membrane	SKF96365	Inhibitor	TRPV2 promotes osteoclast differentiation	[13]
	TRPV4	Plasma membrane	–	–	TRPV4 activation promotes osteoclast differentiation and bone resorption	[60]
TRPMs	TRPM7	Plasma membrane	–	–	TRPM7 regulates MM cell motility and dissemination	[68]
GPCRs	CaSR	Plasma membrane	CaCl_2_ Gadolinium Neomycin	Activator Activator Activator	CaSR promotes the mitosis of MM cells	[57]
	CTR	Plasma membrane	Calcitonin	Activator	CTR inhibits bone resorption by neutralizing OC migration and shape retraction, and may participate in the osteoclast differentiation of MM	[85] [89]
	PTH-R1	Plasma membrane	PTHrP	Activator	PTHrP stimulates the secretion of PTH-R1, promotes proliferation of MM cells and the production of osteoclastogenesis factors	[90] [92]
Purinergic receptors	P2X7	Plasma membrane	ATP	Activator	Activation of P2X7 may induce the apoptosis and prevent the proliferation of MM cells	[15]
SOCE	Stim1	Plasma membrane,	SKF-96365 DES 2-APB	Inhibitor Inhibitor Inhibitor	Silencing Stim1 reduces cell viability, leading to apoptosis and cell cycle arrest of MM cells, and the high expression of Stim1 affects the clinical outcome of MM. In addition, Stim1 could regulate the motility and dissemination of diffuse large B-cell lymphoma (DLBCL) cells and MM cells	[16] [68] [126]
		Endoplasmic reticulum				
	Orai1	Plasma membrane,	SKF-96365 DES 2-APB	Inhibitor Inhibitor Inhibitor	Silencing Orai1 reduces cell viability, leading to apoptosis and cell cycle arrest of MM cells. And Orai1 regulates the motility and dissemination of diffuse large B-cell lymphoma (DLBCL) cells and MM cells	[16] [68] [111] [126]
		Cytosol	AnCoA4			
	TRPC1	Plasma membrane	–	–	Knockout of TRPC1 inhibits the death of MM cells	[132]
Mitochondrial Ca^2+^ transporters	VDAC1	Mitochondrion, Nucleus	–	–	VDAC1 promotes the growth of MM cells, accelerates the development of MM, and affects the prognosis of patients	[17] [144]
	MCU	Mitochondrion	Ruthenium red	Inhibitor	MCU can reduce MM bortezomib resistance and promote MM cell apoptosis	[151] [152]

## Plasma membrane Ca^2+^ channels

### TRP channels

The TRP superfamily channels consist of many non-selective cation channels, including more than 30 members and can be further divided into seven subgroups, i.e. TRPV (vanilloid), TRPC (canonical), TRPM (melastatin), TRPML (mucolipin), TRPP (polycystic), TRPN (no mechanoreceptor potential C), and TRPA (ankyrin) [18]. Each subgroup contains several channel subtypes, which have different Ca^2+^ selectivity, activation mechanisms and interacting proteins [19]. These channels consist of six transmembrane domain segments (S1–S6) and intracellular carboxy (C-) and amino (N-) termini in the pore region between S5 and S6 [20]. TRP family is one of the leading calcium channels [21], which regulates intracellular Ca^2+^ concentration and plays a vital role in various physiological functions, including mediating pain transmission, bone metabolism, and tumor occurrence and metastasis [22, 23]. For example, the TRPV family is related to the onset and progression of MM and chronic myeloid leukemia [24]. The TRPM family, especially TRPM7, has been shown to regulate B cell development and antigen recognition [25]. The TRPC family is involved in the occurrence and development of acute T-cell leukemia tumors [26]. Next, we aim to outline the role of TRPV and TRPM ion channels in MM, and the role of TRPC in MM will be introduced in the part of endoplasmic reticulum SOCE.

TRPV channel structures have six highly conserved transmembrane domain architecture. The functional TRPV channels are tetrameric assemblies that surround a central permeation pathway [27]. The six members of TRPV family can be further divided into three distinct groups based on functional characteristics: fairly non-selective cation channels TRPV1-3 [28], cation-selective efflux channel TRPV4 [29], and epithelial channels TRPV5 and TRPV6 [30, 31]. In TRPV family, studies have reported the importance of TRPV1, TRPV2, and TRPV4 in MM.

TRPM family is the largest and most diverse subfamily in the TRP superfamily [32], composed of eight members, TRPM1 to TRPM8. The TRPM channels have a large cytosolic domain with 732–1611 amino acids for per subunit [33]. Most of the TRPM channels are non-selective Ca^2+^ permeable cation channels; only TRPM4 and TRPM5 are impermeable to Ca^2+^[34, 35]. In this part, we mainly discuss the role of TRPM7 in MM tumorigenesis.

#### TRPV1 channel

TRPV1 is the first member of the TRPV family, which respond to low pH value (< 5), capsaicin noxious heat (> 43 °C) and so on [36, 37]. TRPV1 was initially discovered in rat dorsal root ganglion [38], and involved in the regulation of calcium signaling, inflammation and metabolism, and was closely related to cancer [39, 40]. The functional expression of TRPV1 has been confirmed in several human malignancies including breast, prostate, urothelial cancer and glioma [41–44], yet remained largely unknown in hematologic malignancies especially in MM. At present, drug resistance remains a major challenge for MM cure and calcium signaling was proposed to play a role in drug resistance of cancer cells [45]. TRPV1 has been recognized as an important regulator of intracellular calcium levels, and it was found to be highly expressed in MM cell lines (RPMI8226, INA6 and MM.1S) in a chip analysis of GSE5900 and GSE2658. Recently, a study proposed the role of TRPV1 in MM tumor progression and bortezomib resistance. It is known that induction of endoplasmic reticulum (ER) stress is one of the main mechanisms of bortezomib-mediated cell death. In response to ER stress conditions, the unfolded protein response (UPR) signaling cascade is activated to counteract the occurring damage [46]. Beider et al. found that TRPV1 inhibition (using a pharmacological inhibitor AMG9810) resulted in calcium-dependent accumulation of mitochondrial reactive oxygen species (ROS), followed by mitochondrial instability and MM cell death [47]. These results are consistent with previous findings, indicating that calcium is a key regulator of mitochondrial function, and that calcium overload can impair electron transport leading to ROS generation [48]. In addition, TRPV1 inhibitor (AMG9810) interferes with calcium signaling and suppresses chemokine receptor CXCR4 (CXC-Motif Receptor 4)-mediated migration and stromal protection. It acts synergistically with bortezomib to target ubiquitin pathway and cytoprotective mitochondrial UPR, impairs mitochondria, destabilizes lysosome and promotes MM cell death. On the contrary, TRPV1 agonist (capsaicin) promotes calcium influx, resulting in transient increase in cytosolic calcium levels, thus supporting CXCR4-mediated activity [47]. Importantly, the combination of TRPV1 inhibitor (AMG9810) and bortezomib showed superior anti-MM activity in vivo model of CXCR4-driven human MM engrafting in murine bone marrow [47]. Altogether, these results reveal the mechanism mediating the synergistic anti-MM activity of bortezomib in combination with TRPV1 inhibition which may be translated into clinical practice.

#### TRPV2 channel

TRPV2 is widely expressed in different cells and tissues [49], such as B lymphocyte [50], CD^34+^ hematopoietic stem cells [51], and is a Ca^2+^ permeable channel that contributes to calcium homeostasis [28]. TRPV2 expression in some tumor cells is significantly higher than that in normal cells [52–54], and according to the Cancer Cell Line Encyclopedia (CCLE) database, the expression of TRPV2 is higher in MM cell lines compared to other tumor cell lines. In the study of TRPV2 expression, the protein was detected to be highly expressed in MM patient and MM cell lines (ARP-1, LP-1) by Immunohistochemistry and Western-blot analysis technology, indicating that TRPV2 played a role in MM development [13]. Numerous studies have shown that TRPV2 expression is related to tumor prognosis. In bladder cancer, TRPV2 promotes tumor cell migration and invasion through metalloproteinase 2 (MMP2) [55]; in oesophagal squamous cell carcinoma, high expression of TRPV2 has confirmed to be related to the patient’s disease stage and overall survival [56]. Bai et al. analyzed public gene expression data of bone marrow plasma cells from GSE24080. They found that MM patients with asymptomatic survival and overall survival less than 24 months, the transcriptional level of TRPV2 was significantly higher than that in patients with approximately 24 months [13]. In addition, TRPV2 is related to the occurrence of bone lesions in MM. Myeloma cells often expose to high levels of extracellular calcium concentrations (Ca_o_^2+^) in the microenvironment surrounding destructive bone lesion [57]. Laboratory studies have shown that a high concentration of Ca_o_^2+^ could increase the expression of TRPV2 in ARP-1 and LP-1 cell lines. TRPV2 up-regulates intracellular calcineurin protein, promoting the dephosphorylation of NFATc3 and accelerating NFATc3 to enter the nucleus, thereby enhancing the synthesis and secretion of osteoclast-related factors (such as Receptor Activator of Nuclear Factor-κ B Ligand (RANKL)) in MM cells, ultimately leading to MBD [13]. SKF96365, an inhibitor of TRPV2, proved to be able to inhibit RANKL-mediated osteoclast differentiation, suggesting that SKF96365 may be used as a new type of treatment for MBD [13].

#### TRPV4 channel

TRPV4, a Ca^2+^‐permeable channel of the TRP family, regulates the homeostasis of intracellular calcium concentrations (Ca_i_^2+^) [58]. It mediates Ca^2+^ influx in the late stage of osteoclast differentiation, thus regulating Ca^2+^ signal, which is crucial for cellular events during osteoclast differentiation [59]. MM patients often accompany by abnormal bone metabolism, and the occurrence of MBD is related to the differentiation of osteoclasts [13]. Studies have shown that TRPV4 gene knocked out mice increase bone mass by impairing bone resorption, and TRPV4 activation promotes osteoclast differentiation and bone resorption [60]. In recent years, autophagy has been reported not only as an essential mechanism for maintaining cell homeostasis but also play a vital role in the regulation of osteoclastogenesis [61]. Cao and his colleagues found that TRPV4 up-regulated autophagy-related proteins and Ca^2+^-calcineurin-NFATc1 signaling, thereby promoting the secretion of osteoclast-related factors and modulating osteoporosis [14]. In summary, TRPV4 can be used as a potential therapeutic target for MBD and other bone resorption diseases.

#### TRPM7 channel

TRPM7 is an ion channel that regulates cellular magnesium and calcium homeostasis [62], though it specifically promotes Ca^2+^ influx in various cancer cells [63, 64]. TRPM7 is essential for embryonic development, and deletion of TRPM7 in the chicken B cell line DT40 results in growth arrest and cell death [65]. Earlier, Krishnamoorthy et al. demonstrated for the first time that TRPM7 can regulate B cell development and antigen recognition [25], suggesting its role in MM tumorigenesis.

Recently, growing evidence has demonstrated that MM originates from BM and disseminates throughout the body (also called extramedullary MM), which are closely correlated with poor prognosis with an overall survival period of less than 6 months [66, 67]. It is generally believed that the BM microenvironment provides support for MM cell growth and survival and for the acquisition of aggressive phenotypes. High Ca^2+^ and altered Ca^2+^ signals in the BM microenvironment may be a key contributing factor to the pathological process [68]. Therefore, Ca^2+^ channels and transporters, which are molecular participants of Ca^2+^ homeostasis, could be significantly involve with MM progression in term of MM cell motility and dissemination. Samart et al. observed that the expression of Ca^2+^ influx channels (such as TRPM7) in patient-derived MM cells are upregulated compared with normal plasma cells in a bioinformatics database. They used the CRISPR/Cas9 system to repress TRPM7 gene expression in MM cells, which can significantly inhibit the migration and invasion of MM cells [68]. Such result can reduce the intravasation of MM cells from the BM into nearby blood vessels and for their subsequent extravasation into distant tissues [69, 70]. In addition, vitro and vivo experiments show that TRPM7, Orai1 and Stim1 (Orai1 and Stim1 will be described in detail in SOCE) mediated Ca^2+^ influx regulate MM cell motility and dissemination by orchestrating O-GlcNAylation homeostasis that targets integrin α4 and integrin β7 [68]. In summary, TRPM7 is a key regulator of MM cell motility and dissemination, and may be a potential predictive biomarker and therapeutic targets for advanced MM. Further studies on the role of novel TRPM7 small molecule inhibitors in disseminated MM xenograft models in vivo may be beneficial to future MM treatments to achieve long-term disease control.

### G protein-coupled receptors (GPCRs)

GPCRs are the largest family of membrane signaling proteins in the human body. The core structure of all GPCRs are very similar: 7 transmembrane helix domains, extracellular N-terminus, and intracellular C-terminus [71]. There are two extra-membrane loops (Loop) and three intra-membrane loops. The C-terminus and the intracellular loop connecting the fifth and sixth transmembrane helices have binding sites for protein G (guanylate binding protein). Most GPCRs belong to class A [71], which are rhodopsin-like receptors. Class B GPCRs can interact with peptides. Class C GPCRs are dimers composed of two 7 TM units, which are homologous to bacterial proteins involved in the transport of amino acids and ions [72]. And there are no receptors belong to class D, E, F or O in the human genome [71]. GPCRs usually localize on the plasma membrane, which mediates the response of cells to various stimuli, ultimately cause cellular responses [73]. Here, we mainly explain the role of GPCRs in the Ca^2+^ transport process.

#### Calcium sensitive receptor (CaSR)

Calcium-Sensing Receptor (CaSR) is a class C GPCRs discovered in 1993 by Brown and coworkers [74]. It is widely distributed in the human body and participates in numerous physiological and pathological processes, monitoring extracellular Ca^2+^ concentration and responding to various signals stimulate, and then regulating Ca^2+^ homeostasis [74]. CaSR is a homodimer, and each monomer composes an N-terminal extracellular domain (ECD), a seven-transmembrane domain (TTM) and an intracellular C-tail domain (C-tail) [75]. Ca^2+^ is the primary agonist of CaSR and can bind to the ECD region [76], and is thought to be involved in regulating mitotic transitions [77]. Yamaguchi et al. found that CaSR can promote the mitogenic response of mouse osteoblastic MC3T3-E1 cell [78]. CaSR agonists (such as Ca^2+^, gadolinium (Gd^3+^), and neomycin) increased levels of inositol triphosphate (IP3), cytosolic calcium concentration and DNA synthesis through a mechanism coupled to the activation of G protein and PKC [74, 79]. Besides, the mitogen-activated protein kinase (MAPK) family members, p42/44 and p38 MAPK, have been confirmed to be involved in CaSR-stimulated mitogenic response in mouse osteoblastic MC3T3-E1 cell. These responses may serve to ensure the availability of adequate numbers of osteoblastic cells at sites of recent bone resorption [80]. MM often accompanies by an increase in Ca_o_^2+^, which leads to changes of CaSR expression in hematopoietic precursor cells, and ultimately red blood cell precursors, megakaryocytes (the precursors of blood platelets), monocytes, and macrophages, all of which express higher levels of the CaSR than white blood cells precursors [81]. In human myeloma cells, such as U266, IM-9 and RPMI8226 cells, exposure to high Ca^2+^ concentration augmented cell proliferation through CaSR on their surfaces, and further participate in a vicious cycle by expanding myeloma cell mass in destructive bone lesions [57]. Studies have reported that some basal or constitutive activity of MAPK, Interleukin-6 (IL-6), insulin-like growth factor-1 (IGF-1) or other signaling pathways have been found in these myeloma cells, which can promot cell proliferation [82, 83]. This is how myeloma cells survive in bone marrow microenvironment, indicating that CaSR, at least in part, might mediate these survival signals to regulate the mitosis of myeloma cells. However, CaSR activators (such as Ca^2+^, NPS, R467) did not enhance IL-6 expression in these cells, indicating that CaSR agonists exerting a mitogenic effect on myeloma cells seemed to be independent of IL-6 actions [57]. In addition, CaSR agonists have shown to stimulate proliferation of osteoblasts, monocyte-macrophages, bone marrow stromal cells and fibroblasts [57], which further supports the view that CaSR activation can promote MM mitosis.

#### Calcitonin receptor (CTR)

Calcitonin receptor (CTR) belonged to class B GPCR and identified originally in porcine kidney epithelial cell line [84]. CTR primarily expressed in osteoclasts (OCs), K562 myeloid leukemia cells, human breast cancer, kidney, and mouse and human lymphocytes, with a down-regulating effect on its function in response to hypercalcemia [85]. CTR contains seven transmembrane regions, four extracellular loops (ECL1-4) and four intracellular loops. It has proved that the ECL2 and ECL3 region are the binding sites of calcitonin (CT) [86]. Six structural variants have described, i.e. CTR-1 to -6 isoforms with most unknown functions. CTR structural isoforms can activate several heterotrimeric G proteins in response to CT stimulation, trigger various signal transduction mechanisms, that lead to intracellular Ca^2+^ isoform or adenylate cyclase accumulation throughout PKC or PKA pathway, respectively [87]. The CTR-2 isoform was detected on Ocs, with 16 amino acid deletions in the first intracellular loop [88]. Its main functions include the inhibition of bone resorption by neutralizing OCs migration and a shape retraction [89]. Silvestris et al. have shown that myeloma cell lines (U-266, MCC-2 and RPMI 8226 cells) express CTR-2 isoform and activate both cAMP and Ca^2+^ flow, suggesting the activation of PKA and PKC pathways. In addition, in myeloma cells, CT effectively inhibits bone resorption in vitro, supports the OC-like behavior of myeloma cells, indicating that CT or CTR may participate in the osteoclast differentiation of MM [85].

#### Parathyroid hormone-related protein Receptor (PTH-R1)

Parathyroid hormone-related protein (PTHrP) is functionally similar to parathyroid hormone (PTH), which is the main regulator of Ca^2+^ homeostasis and an OC activator. The NH2 terminal fragment of PTHrP can bind to PTH-R1, resulting in a significant increase in intracellular Ca^2+^ influx [90]. PTH-R1 is mainly distributed in bones and kidneys, and belongs to class B GPCRs. The receptor has seven transmembrane regions, longer extracellular amino acids, and contains more than three pairs of disulfide bonds folded into a network. The extracellular region cooperates with the helical structure of the transmembrane area and participates in the binding of ligands, and PTHrP is the only known PTH-R1 endogenous ligand [91]. There is little information about the role of PTH-R1 in MM. Previous studies reported that PTH-R1 highly expressed in bone marrow plasma cells of MM patients and MM cell lines, and PTHrP stimulated PTH-R1 in MM cells, promoting the proliferation of MM cells, and eventually the increase of Ca^2+^ influx, CAMP content [92]. Cafforio used ELISA and flow cytometry to demonstrated that in U266, MUS and RPMI 8226 cells, PTH-R1 directly participated in reinforcing both RANKL and monocyte chemoattractant protein 1 (MCP-1) production, while the opposite effect could be observed after PTH-R1 silenced [92]. The above results have clarified that PTHrP promotes MM tumor biological behavior and development through autocrine or paracrine stimulation of PTH-R1, thereby reinforcing the production of osteoclastogenic factors. In addition, they further verified that the PTHrP NH2 fragment (aa 1–40) reacted with PTH-R1 in RPMI 8226 cells, but not the NLS-middle region (aa 67–86), NLS middle region (aa 38–94) and COOH end (aa 107–139) fragments. PTH-R1 systematic analysis is still needed in MM patients to determine whether the receptor is a new marker of disease progression.

### Purinergic receptors

Purinergic receptors, namely P1 and P2 receptors, are widely expressed in mammalian cells and are involved in several physiological functions like nociception, platelet aggregation, inflammatory reaction etc. [93]. The P2 receptors are divided into two groups: P2X and P2Y, each containing some members with different ion selectivity and regulatory properties. The P2X receptors have a trimeric topology with two transmembrane domains. The P2X7 receptor is a member of P2X, mainly expressed in bones, epithelial cells and normal B lymphocytes [94]. ATP can activate P2X7, leading to the absorption of cations such as Ca^2+^, Na^+^ and K^+^[95]. Low activation of P2X7 receptor promotes cell division, while an acute stimulation by massive ATP causes cell death [96]. For instance, activation of P2X7 in chronic lymphocytic leukaemia (CLL) results in cell death [97, 98] and induces the rapid release of cell-surface CD23 from CLL B lymphocytes [99]. CD23 is a ‘low affinity’, transmembrane receptor for IgE that can be shed from the cell surface to form soluble CD23 (sCD23), which also binds IgE and exerts cytokine-like activities on B cells and other leukocytes [100]. sCD23 maintains B-cell precursors growth, promotes B and T cell differentiation, and drives monocytes to release cytokines [101]. Compared with the plasma sCD23 levels of normal individuals, the sCD23 concentration of CLL patients may be 5 to 300-flod higher [102]. In addition, individuals with low plasma sCD23 concentrations have a more positive prognosis than those with high sCD23 concentrations [103].

Although P2X7 receptor is playing a role in neoplasia, long-term lack of malignant cell lines expressing this receptor, resulting in progress limited of P2X7 receptor in tumorigenesis. In recent years, Farrell et al. found functional P2X7 on the RPMI 8226 MM cell line. In this studies, P2X7 activation-induced death of RPMI 8226 cells, prevented the proliferation of RPMI 8226 cells and also generated CD23 shedding from myeloma cells [15]. As a useful marker in either diagnosis or prognosis of disease [104–106], sCD23 can provide useful value for further study of the mechanism of P2X7 in myeloma. In addition to P2X7, RT-PCR also showed that P2X4 and P2X5 are high expressions and P2X1 is low expression in RPMI 8226 cells, but it is still unknown whether these mRNAs are translated to proteins or lead to functional P2X channels [15]. At present, the physiological and pathophysiological effects of P2X in myeloma remain unclear and requires further research to elucidate.

## Store-operated Ca^2+^ entry (SOCE)

Store-Operated Ca^2+^ Entry (SOCE), as a calcium ion channel, has been widely recognized as one of the major pathways for Ca^2+^ influx across PM of cells [107, 108]. The main components of SOCE are Stim proteins and Orai proteins (also known as CRAC). Stim proteins, located in the ER, are single-pass transmembrane proteins. Their EF-hand motif of N-terminal domain serves as a Ca^2+^ sensor within the ER [109]. In response to ER Ca^2+^ depletion, Stim proteins undergo a conformational change, forming self-aggregation and CRAC channels at the ER-PM junction through a diffusion trap mechanism, which promotes Stim proteins binding and activating hexamers of the Orai pore-forming proteins to trigger Ca^2+^ entry [110]. Stim proteins have two subtypes, Stim1 and Stim2, and there are three subtypes of Orai proteins in mammals, namely Orai1, Orai2 and Orai3 [110]. Orai proteins are four-time transmembrane membrane proteins, and their N-terminus and C-terminus are both in the cytoplasm, and TM1 forms the pore of the ion channel. Stim and Orai proteins widely expressed in various organs and tissues in the human body. Their deficiency or malfunction can lead to alterations in Ca^2+^ handling and ultimately cause oncogenic transformation [111]. In addition, the TRPC channel has suggested to be a component of SOCE channels [112]. It has reported that Orai1 is indispensable to TRPC1 function through the dynamic signal complex composed of Stim1- Orai1- TRPC1 [113, 114]. At rest, TRPC1 docked in the trafficking vesicle of the PM. After the storage is depleted, TRPC1 inserts into the PM, which is then gated and activated by Stim1, allowing TRPC1 mediated Ca^2+^ influx. TRPC1 plays a vital role in the overall function of the SOCE pathway [111]. Altered SOCE activity or the remodelling of SOCE-expression profile have reported in numerous cancers, such as colorectal cancers [115], hepatocellular carcinoma [116], melanoma [117], breast cancer [118], glioblastoma [119] and clear cell renal carcinoma [120]. Until now, tumor cell migration is considered as a Ca^2+^ dependent process, and calcium channels are major regulators of this process [121]. In non-excitable cells, calcium entry is mainly due to SOCE. Orai1 and Stim1 are the main calcium channels responsible for Ca^2+^ entry in lymphocytes [122, 123], and both have been described to be involved in the mediation of actomyosin assembly and the focal adhesions of cancer cell migration [124, 125]. Latour el al have demonstrated that Orai1 and Stim1 could regulate diffuse large B cell lymphoma (DLBCL) cell motility and dissemination, promoting homing of tumor B cells to extra-nodal sites [126]. Although in-depth researches have conducted, little is known about the role of SOCE in MM. Stim1 and Orai1 are the critical components of CRAC channels that highly expressed in MM bone marrow tissues. And compared with healthy controls, the mRNA levels and protein level of Stim1 and Orai1 were higher in primary MM cells, KM3 and U266 cells [16]. Moreover, U937 and RIMP 8226 MM cell lines also express higher levels of Orai3 [127]. Wang et al. demonstrated that SOCE inhibitors (SKF-96365, DES and 2-APB) affect the biological functions of human MM cell lines, not only inhibited the viability of MM cell lines, caused MM cell cycle arrest, but also induced MM cell apoptosis. Consistently, pretreatment of KM3 and U266 cells with the siRNAs of Stim1 or Orai1 for 48 h also reduced cell viability, leading to apoptosis and cell cycle arrest in MM cell lines, indicating that SOCE plays a role in MM development [16]. Moreover, clinical data found that Stim1/Orai1 highly expressed in the stage III group than stage II and I group in MM. Progression-free survival (PFS) (15.33 months) of patients in low Stim1 expression was greater than that of PFS (13.34 months) high Stim1 expression group [16]. The above results indicate that Stim1/Orai1 participates in the pathogenesis of MM, but the mechanism by which it works requires further research.

As a potential regulator of SOCE, TRPC1 widely expressed in most tissues, and its dysregulated activity may be a hallmark of many types of cancers, including glioblastoma multiforme [128], breast cancer [129], colon cancer [130], multiple myeloma [131]. Some studies on vitro mechanisms of TRPC1 inducing an increase in intracellular Ca^2+^ in the MM cell lines, indicating that TRPC1 is related to the development of MM. Knockout of TRPC1 expression showed a reduction in MTI-101(an frist-in-class peptidomimetic that triggers cell death in MM)-induced cell death in U266 and MM.1S MM cell lines [132]. This study suggests that TRPC1 plays an important role in treating MM. However, there is still needed to develop more specific drugs to inhibit or activate TRPC1 function, so that the efficacy of TRPC1 as a potential target for cancer treatment can be verified.

## Mitochondrial Ca^2+^ transporters

Mitochondria have shown to have a significant contribution to the activity of SOCE, and the ability to chelate Ca^2+^ locally at the sites of Ca^2+^ release and entry was supposed to rule SOCE by promoting Ca^2+^ depletion of the ER and removal from Ca^2+^ inhibitory channels, respectively [133]. The uptake of mitochondrial Ca^2+^ must be precisely regulated, because excessive mitochondrial Ca^2+^ load can cause cell death [134]. Several proteins mediate Ca^2+^ transport across internal and outernal mitochondrial membranes (IMM, OMM, respectively), including VDAC1 on OMM, and MCU and Na^+^ dependent mitochondrial Ca^2+^ efflux transporter (NCLX) on IMM. Ca^2+^ is transferred to the IMM through OMM. VDAC1 allows Ca^2+^ to enter MCU, thus promoting Ca^2+^ transport to the matrix and also from IMM to cytoplasm [11]. This section focuses on VDAC1 and MCU function in the uptake and release of mitochondrial Ca^2+^ and their effects on MM.

### Voltage-dependent anion channel 1 (VDAC1)

VDAC1 is located on the outer membrane of mitochondria in all eukaryotes and is a pore type protein. VDAC1 is structurally organized into a transmembrane β-bucket with an N-terminal domain as an α helix [135]. It acts as a voltage-gated channel to control the exchange of small ions and metabolites on the OMM, maintaining many organelle functions [136, 137]. VDAC1 is the only known Ca^2+^ transporter on OMM [11, 138], which leading to mitochondrial Ca^2+^ overload, is a marker of cell apoptosis [139]. Recent studies have shown that apoptosis signal up-regulates VDAC1 in a Ca^2+^ dependent manner, leading to cell death [140, 141]. It is now clear that VDAC1 is the main target against cancer. Compared with normal cell lines, the expression of VDAC1 increases in much human cancer cell lines [142]. However, research on the effect of VDAC1 on MM cell apoptosis is still relatively rare. Previous studies have indicated that the expression of CD45 in human myeloma cells is necessary for IL-6-induced proliferation [143]. Liu et al. found that susceptibility to apoptosis by apoptotic stimulis (such as oxidative stress and ER stress) are increased in CD45^+^ myeloma cell line U266, which highly expressed VDAC1 [17]. Together with the information that expression of VDAC1 in patients with stage III was significantly higher than that in patients with stage I and stage II [144], one could imply that VDAC1 seems to accelerate MM tumor growth, while increase susceptibility to apoptosis, making it a good target candidate for MM therapy.

### ***Ca***^***2***+^***uniporter (MCU)***

In IMM, the rapid absorption of Ca^2+^ is mediated by the MCU. MCU can actively shape Ca^2+^ signaling throughout the cell [145, 146]. MCU is composed of two transmembrane and N-terminal domains and forms a complex in the IMM with multiple protein regulators, which have an impact on gating [147–149]. It is known that the disregulation of intracellular Ca^2+^ is among the first hallmarks of apoptosis [150], and some reports indicate that MCU plays an important role in bortezomib-induced apoptosis [151, 152]. Bortezomib is a first-in-class selective PI (proteasome inhibitor), which has been proven to be effective in the treatment of MM. However, most patients eventually develop disease recurrence, and the bortezomib resistance becomes the primary cause of recurrence and incurability of myeloma [153]. Therefore, there is an urgent need to identify the potential mechanism of bortezomib resistance, and find some novel therapeutic targets that can reduce bortezomib resistance in MM. Song et al. found that mitochondrial activity is a determining factor in regulating apoptosis resistance in response to bortezomib, and compared with bortezomib-resistant KMS20 cells, the expression of MCU in bortezomib-sensitive KMS28BM cells were significantly higher under bortezomib stimulation [152]. Furthermore, the cytotoxicity of bortezomib against myeloma cells H929, U266 and MM.1S can be inhibited by mitochondrial Ca^2+^ uptake inhibitor ruthenium red (a cationic dye that blocks MCU) [151], indicating that changes in MCU were related with the resistance of bortezomib**.** Landowski's team proposed a model in which bortezomib evokes an instantaneous release of Ca^2+^ stores, leading mitochondrial Ca^2+^ influx. Mitochondrial Ca^2+^ sensors associated with uniporters initiate capacitative Ca^2+^ influx, also called CCI, a phenomenon that IP3R-activated depletion of ER stores is also involved in the regulation of Ca^2+^ influx from the extracellular environment [154], which is enhanced in bortezomib-treated cells, leading to apoptotic pathways activation [151]. Then, MUC blocker would be protective by blocking mitochondrial loading and CCI activation and the Ca^2+^-dependent signal transduction pathways that initiate cell death [155]. Together, MCU is expected to become a new therapeutic target to reduce MM bortezomib resistance. However, the specific mechanism of MUC participation in resistance still needs intense investigation.

## Ca^2+^-ATPases

Ca^2+^-ATPases, which belong to the P-type pump superfamily, are involved in maintaining low cytoplasmic Ca^2+^ levels at rest and initiating organelle stores [156]. They quickly pump cytosolic Ca^2+^ ions back to intracellular organelles, such as ER, or to squeeze Ca^2+^ ions into extracellular space [10]. According to their subcellular location, Ca^2+^-ATPases can be divided into three subtypes: plasma membrane (plasma membrane Ca^2+^ -ATPase or PMCA), endoplasmic reticulum (Sarco/endoplasmic reticulum Ca^2+^-ATPase or SERCA) and Golgi apparatus / Golgi-derived vesicles (secretory pathway Ca^2+^-ATPase or SPCA) [157] (Fig. 1). Among them, SERCA is the most distinctive one, which is responsible for replenishing ER Ca^2+^ storage and maintaining correct folding of proteins. SERCA imbalance leads to decreased or overloaded Ca^2+^ storage in the ER lumen, increased ER stress, chaperones protein imbalance and lipid synthesis [10, 158]. Their expression levels and tissue distribution in the body are different [159]. Mutations and changes in SERCA subtypes expression levels have confirmed in a variety of cancers, such as colon cancer, gastric cancer, lung cancer, myeloid leukemia, and choroid plexus tumors [160]. Roti and his colleagues showed that SERCA inhibition preferentially damages the maturation of leukemia-related mutant NOTCH receptors, leading to G0/G1 arrest [161]. PMCA is a calcium pump that relies on ATP to drive Ca^2+^ out of the cell. When PMCA expression is abnormal, it will cause the disorder of Ca^2+^ balance in the cell. Studies have found that plasma membrane PMCA is abnormally expressed in various malignant tumors, including breast cancer [162], colon cancer [163], pancreatic cancer [164], and so on. PMCA proved to be a part of the Ca^2+^ exclusion system, but the role of each subtype of PMCA has not studied extensively. The main reason is the lack of specific inhibitors of each subtype of PMCA. The third newly discovered subtype is SPCA, a freshly discovered subtype, include calcium pumps located in the Golgi compartments and post-Golgi vesicles [156]. Changed expression of SPCA subtypes occurs in various types of cancer including breast, prostate and colon cancer [165]. The results of the above studies have shown that Ca^2+^ -ATPase is used to promote the escape of specific cancer cells from normal cellular control and accelerate tumorigenesis. Although clear evidence linking Ca^2+^-ATPases to several malignancies, rare reports about the study of Ca^2+^-ATPase in MM. Further study the role of Ca^2+^-ATPase in MM will contribute to understanding the complex intracellular Ca^2+^ signaling network in MM.

## Conclusion

Calcium channels and transporters play a vital role in the regulation of Ca^2+^ transport and participate in multiple physiological and pathological processes, including the progression of cancer. Although various studies have reported the role of ion transporters in MM, the mechanism by which Ca^2+^ imbalance caused by defective ion transporter function leads to the occurrence of MM remains unclear. Therefore, it is particularly significant to elucidate the aetiology and pathogenesis of MM and explore new tumor markers for early diagnosis and treatment. In this review, we concerned about the effects of various calcium channels and transporters abnormalities on the development of MM, including many plasma membrane Ca^2+^ channels, SOCE, Mitochondrial Ca^2+^ transporters and Ca^2+^-ATPases. This review aims to stimulate people's interest in and to provide a basic, systematic overview of the research in this field, thereby providing new directions for the prognosis and treatment of MM by targeting Ca^2+^ channels or transporters.

## Abbreviations

Ca_o_^2^^+^: Extracellular calcium concentrations
Ca_i_^2^^+^: Intracellular calcium concentrations
CaSR: Calcium sensitive receptor
CCLE: Cancer cell line encyclopedia
CCI: Capacitative Ca^2^^+^ influx
CLL: Chronic lymphocytic leukaemia
CT: Calcitonin
CTR: Calcitonin receptor
DLBCL: Diffuse large B cell lymphoma
ECD: Extracellular domain
ER/SR: Endoplasmic/sarcoplasmic reticulum
GA: Golgi apparatus
GPCRs: G protein-coupled receptors
IMM: Internal mitochondrial membranes
IP3R: Inositol 1,4,5-triphate receptor
IGF-1: Interleukin-6 (IL-6), insulin-like growth factor-1
MAPK: Mitogen-activated protein kinase
MBD: Myeloma bone disease
MCP-1: Monocyte chemoattractant protein 1
MCU: Mitochondrial Ca^2^^+^ uniporter
Mit: Mitochondrial
MM: Multiple myeloma
MMP2: Metalloproteinase 2
NCLX: Na^+^ dependent mitochondrial Ca^2^^+^ efflux transporter
OCs: Osteoclasts
OMM: Outernal mitochondrial membranes
IMM: Internal mitochondrial membranes
PFS: Progression-free survival
PM: Plasma membrane
PMCA: Plasma membrane Ca^2^^+^ -ATPase
PTH: Parathyroid hormone
PTH-R1: Parathyroid hormone-related protein Receptor
PTHrP: Parathyroid hormone-related protein
RA NKL: Nuclear factor-κ B ligand
RyR: Ryanodine receptor
ROS: Reactive oxygen species
SOCE: Store-operated Ca^2^^+^ entry
SERCA: Sarco/endoplasmic reticulum Ca^2^^+^-ATPase
STAT3: Activator of transcription 3
Stim1: Stromal-interaction molecule1
SPCA: Secretory pathway Ca^2^^+^-ATPase
TTM: Transmembrane domain
TRP: Transient receptor potential channel
TRPV: Transient receptor potential channel subfamily V
UPR: Unfolded protein response
VDAC1: Voltage-dependent anion channel 1
